# Host Modulation Through Next-Generation Oral Probiotics: Can Targeted Microbiome Modulation Reverse the Inflammatory State in Periodontitis? A Narrative Review

**DOI:** 10.7759/cureus.117443

**Published:** 2026-10-04

**Authors:** Josue Santoyo, Sandra Morales Corredor, Dejerly Parra Pinto, Yan Diaz Villa, Paola V Castillo Ordonez, Luisana S Rodriguez

**Affiliations:** 1 Dentistry, Ibero-American University, Santo Domingo, DOM; 2 Dentistry, San Martín University Foundation, Bogota, COL; 3 Dentistry, University of Carabobo, Valencia, VEN; 4 Dentistry, Sancti Spíritus Faculty of Medical Sciences, Sancti Spiritus, CUB; 5 Dentistry, Private Practice, Middletown, USA; 6 General Dentistry, Boston University School of Medicine, Boston, USA; 7 Dentistry, Universidad de Carabobo, Valencia, VEN

**Keywords:** dysbiosis, host modulation therapy, immunomodulation, next-generation probiotics, oral microbiota, periodontitis, probiotics, scaling and root planing

## Abstract

Periodontitis is driven less by bacterial load than by a dysbiotic subgingival microbiome acting alongside a dysregulated host response, which together destroy connective tissue and alveolar bone. This understanding has shifted attention to next-generation oral probiotics as a way to target both the microbial and inflammatory components of disease. This narrative review evaluates current evidence on next-generation oral probiotics as a precision approach to periodontal microbiome modulation, their influence on host inflammatory responses and periodontal homeostasis, and their potential to resolve dysregulated inflammation and improve clinical outcomes. We searched PubMed and Google Scholar for systematic reviews, narrative reviews, randomized clinical trials, and observational and experimental studies published between 2021 and 2026. Probiotic supplementation, particularly when combined with conventional therapy such as scaling and root planing, has shown potential to reduce periodontal inflammation and improve selected clinical parameters. Probiotics have been associated with changes in pro-inflammatory mediators, including interleukin (IL)-1β, IL-6, and tumor necrosis factor-alpha (TNF-α), and matrix metalloproteinases, while potentially promoting anti-inflammatory mechanisms involving IL-10 and the resolution of inflammation; additional effects may include modulation of oxidative stress, improved epithelial barrier function, and indirect regulation of osteoclast-mediated alveolar bone remodeling. Current evidence nonetheless remains heterogeneous in strain, dose, and protocol, with mostly short follow-up, so probiotics are best regarded as a promising adjunct that attenuates inflammation and enhances conventional therapy rather than an independent means of reversing established periodontitis. Standardized, strain-specific protocols and adequately powered long-term trials are needed.

## Introduction and background

Periodontitis is a chronic inflammatory disease of the tooth-supporting tissues in which a dysbiotic subgingival microbiome (a disease-associated shift in microbial composition and function away from a health-associated community) and a dysregulated host response together drive the progressive loss of connective tissue attachment and alveolar bone. A central insight of contemporary periodontology is that disease severity is not predicted by bacterial burden alone: individuals harboring comparable organisms differ in outcome according to the intensity, duration, and resolution of their inflammatory response, and most attachment and bone loss is inflicted by host-derived enzymes and osteoclasts rather than directly by bacteria [1-2]. This dual pathogenesis has reframed the therapeutic goal from microbial elimination toward restoring ecological balance while modulating the host response that dysbiosis provokes [3].

Two complementary strategies follow from this view. Host modulation therapy targets the destructive inflammatory response, while microbiome-directed approaches aim to reconstruct a health-associated community rather than sterilize the site. Next-generation oral probiotics (rationally selected or engineered strains designed for defined functions, as opposed to conventional off-the-shelf probiotics) sit at the intersection of both, because they may simultaneously displace pathobionts (commensal organisms that turn harmful under dysbiotic conditions) and attenuate inflammation. This narrative review evaluates current evidence on next-generation oral probiotics as a precision approach to periodontal microbiome modulation, examining their influence on host inflammatory responses and periodontal homeostasis, and their potential to resolve dysregulated inflammation and improve clinical outcomes.

## Review

This structured narrative review was conducted with predefined search terms and explicit selection criteria rather than as a systematic review. We searched PubMed/MEDLINE and Google Scholar for records published between January 2021 and 2026, using combinations of the terms periodontitis, periodontal disease, dysbiosis, subgingival microbiome, host modulation therapy, probiotics, next-generation probiotics, postbiotics, Lactobacillus reuteri, Streptococcus salivarius, scaling and root planing, interleukin, matrix metalloproteinase, and receptor activator of nuclear factor kappa-B ligand (RANKL)/ osteoprotegerin (OPG). We considered systematic reviews, meta-analyses, randomized controlled trials, and observational and experimental studies, and hand-searched the reference lists of retrieved articles for additional records. Seminal earlier work was retained where it established a foundational concept still in use, such as the keystone-pathogen hypothesis and the oral-microbiota framework, and is identified as foundational rather than contemporary evidence. Records were screened by title and abstract and assessed in full text by the authors, with disagreements resolved by discussion. Consistent with a narrative design, no formal risk-of-bias appraisal, Preferred Reporting Items for Systematic Reviews and Meta-Analyses (PRISMA) flow diagram, or quantitative synthesis was undertaken.

Periodontal dysbiosis and host response: from microbial imbalance to chronic inflammation

The healthy periodontium is not sterile but stable. Hundreds of bacterial species occupy distinct supragingival and subgingival niches, and their collective activity supports colonization resistance and immune tolerance rather than damage [1]. Health-associated communities are dominated by facultative and aerotolerant genera such as Streptococcus, Actinomyces, and Rothia, and the equilibrium they maintain depends on signaling among community members and on host factors including salivary flow, crevicular fluid composition, and epithelial barrier integrity [3]. Disease begins when this balance is lost. Dysbiosis is best understood as a functional and compositional shift, with loss of beneficial commensal diversity and expansion of anaerobic, proteolytic, inflammophilic species, rather than as the arrival of a single infectious agent [1,3].

The keystone pathogen hypothesis explains how such a shift can be orchestrated by an organism present in very low numbers. Porphyromonas gingivalis remains a minor constituent of the subgingival biofilm, yet it manipulates innate immunity, particularly the crosstalk between complement C5aR1 and Toll-like receptors, so that bacterial clearance is impaired while inflammation persists [4]. More recent work has refined this concept: gingipains, outer membrane vesicles, and lipid A modification allow the organism to escape killing while sustaining an inflammatory environment, and its keystone behavior is strain-dependent and shaped by local conditions [2]. Because a keystone organism cannot act alone, the polymicrobial synergy and dysbiosis model situates it within a community in which accessory species facilitate colonization and metabolic cooperation, while commensals turned pathobionts amplify destruction downstream [1-2]. The result is a feedforward loop: inflammation releases degraded proteins and heme; these substrates favor asaccharolytic anaerobes, and their expansion sustains further inflammation [1].

What converts this ecological shift into tissue loss is the host response. Neutrophils dominate the periodontal lesion, and when their recruitment and microbicidal function are dysregulated, degranulation and extracellular trap release spill proteases and reactive oxygen species into the surrounding connective tissue [3]. Adaptive immunity is drawn toward a T helper 17 profile, and interleukin (IL)-17, protective at physiological levels, becomes pathological when persistently elevated, driving further neutrophil recruitment, RANKL expression, and matrix metalloproteinase (MMP) activity [5]. IL-1β, TNF-α, and IL-6 maintain the loop through nuclear factor kappa-B (NF-κB) and mitogen-activated protein kinase signaling [6]. Equally important is what fails to happen: resolution. When pro-resolving mechanisms do not restore homeostasis, the lesion becomes chronic and self-perpetuating rather than self-limiting [5].

Collagen breakdown in this setting is largely host-derived. MMP-8, the neutrophil collagenase, degrades type I and III collagen, and salivary MMP-8 and its activated form are consistently higher in patients with periodontitis than in healthy controls, supporting their value as indicators of ongoing collagenolysis [7]. Alveolar bone loss follows a comparable logic. RANKL produced by osteoblasts, fibroblasts, and infiltrating T and B lymphocytes binds RANK on osteoclast precursors and drives nuclear factor of activated T cells c1-dependent differentiation, while OPG competes as a decoy receptor; sustained inflammation raises the RANKL/OPG ratio and tips remodeling toward resorption [6]. The clinical implication is that bacterial burden alone does not predict destruction, and recognizing this dual pathogenesis is what justifies moving beyond microbial elimination toward strategies that restore ecological balance while modulating the response it provokes (Figure 1) [2,6].

**Figure 1:**
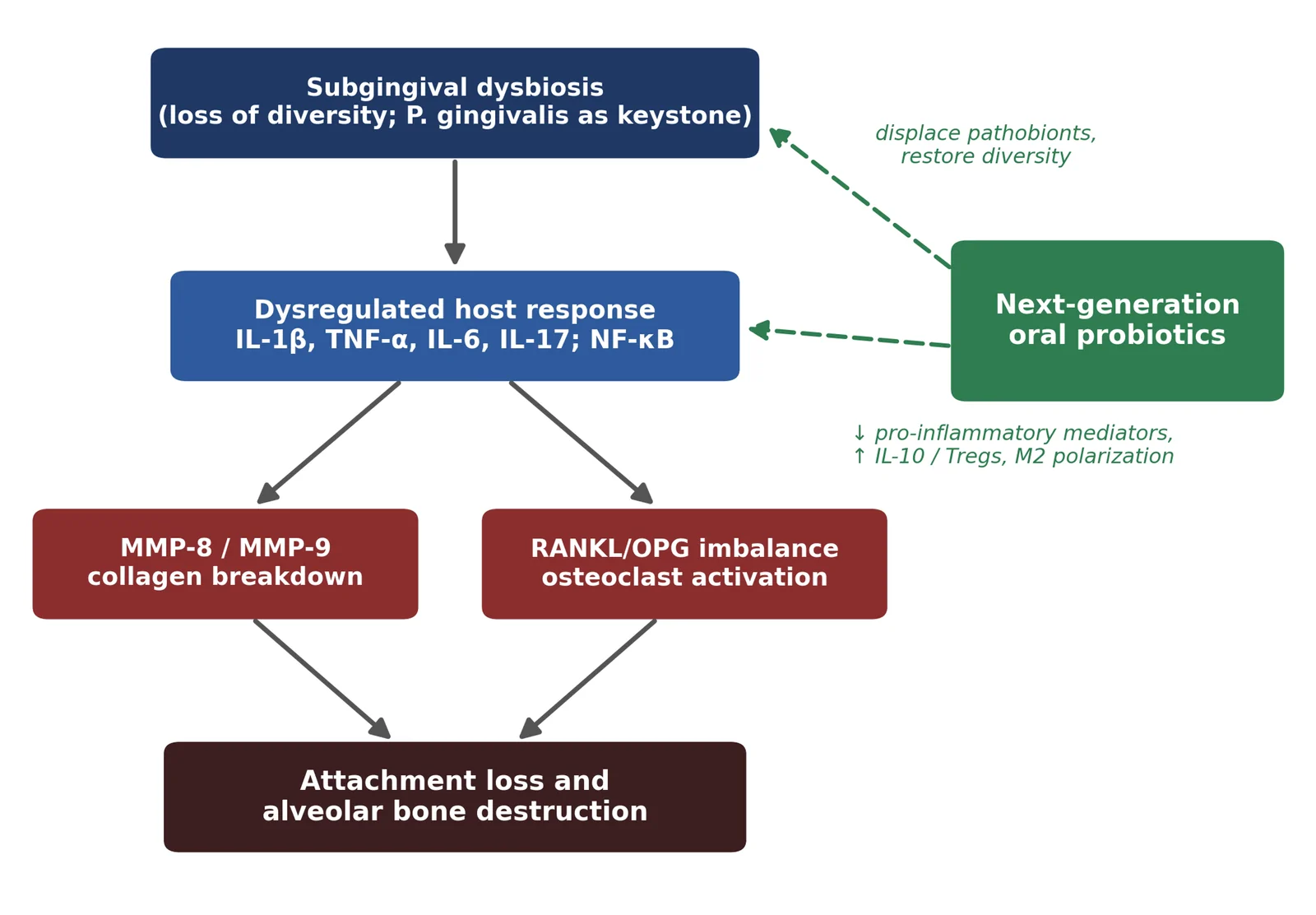
Dual pathogenesis of periodontitis and the two nodes on which next-generation oral probiotics may act. Dysbiosis drives a dysregulated host response that produces host-derived tissue destruction through matrix metalloproteinases and RANKL/OPG imbalance; probiotics may act on both the microbial and inflammatory nodes. IL: interleukin; TNF-α: tumor necrosis factor-alpha; NF-κB: nuclear factor kappa-B; MMP: matrix metalloproteinase; RANKL: receptor activator of nuclear factor kappa-B ligand; OPG: osteoprotegerin.

Host modulation therapy: current concepts and biological targets

Because microbial control alone may not fully address periodontal tissue destruction, a prolonged and dysregulated host inflammatory response contributes to excessive cytokine production, osteoclast activation, and impaired resolution of inflammation [8-9]. This provided the rationale for host modulation therapy (HMT), an approach designed to control destructive host responses while conventional biofilm management is maintained. The concept emerged in the early 1980s and gradually expanded from limiting connective-tissue breakdown to modulating broader inflammatory and bone-resorptive mechanisms [9-10].

Subantimicrobial-dose doxycycline (SDD) is one of the most widely investigated approaches. At subantimicrobial doses, doxycycline acts mainly by inhibiting host-derived MMPs involved in periodontal connective-tissue breakdown [10]. However, its clinical benefits appear relatively small, with only modest improvements in probing depth (PD) and clinical attachment level (CAL) at low or very low certainty of evidence [11]. Omega-3 polyunsaturated fatty acids, particularly eicosapentaenoic acid and docosahexaenoic acid, have also been explored for their anti-inflammatory and pro-resolving properties, and as a complement to periodontal therapy, they have shown potential improvements in PD and CAL, although results vary across studies and protocols [12]. The combination of omega-3 with low-dose aspirin has shown potentially beneficial results, but the available evidence remains limited [11].

Specialized pro-resolving mediators (SPMs), including lipoxins, resolvins, and protectins, offer a different approach: rather than reducing inflammation, these endogenous mediators actively facilitate its resolution and help re-establish tissue homeostasis [13]. Non-steroidal anti-inflammatory drugs reduce inflammation by suppressing cyclooxygenase activity and prostaglandin production, but their prolonged systemic use is limited by adverse effects [13]. Bisphosphonates have been investigated for their ability to inhibit bone resorption, and their use in combination with scaling and root planing has shown additional clinical benefits. However, safety concerns associated with systemic administration limit their use [11,14].

From a biological perspective, NF-κB signaling helps regulate inflammatory mediators, including IL-1β, TNF-α, IL-6, and IL-17, which sustain the inflammatory response [8-9]. MMP-8 and MMP-9 contribute to extracellular matrix and collagen degradation, while RANKL promotes osteoclastogenesis and alveolar bone resorption [9,15]. Human evidence supports these targets: a 2025 systematic review and meta-analysis found significantly elevated salivary levels of IL-1β, MMP-8, and MMP-9 in individuals with periodontitis [15], and more recent meta-analytic evidence identified increased systemic levels of TNF-α, IL-6, IL-17, and RANKL in periodontitis, including analyses restricted to systemically healthy individuals [16]. The principal host modulation strategies and their evidence status are summarized in Table 1. Overall, HMT extends periodontal treatment beyond microbial control, but its clinical application remains limited by modest treatment effects, protocol variability, limited long-term evidence, and safety concerns, which has encouraged the search for more selective strategies that restore inflammatory homeostasis while maintaining a balanced host-microbiota interaction [8,11].

**Table 1:** Host modulation strategies in periodontitis, their principal targets, and current evidence status. MMP: matrix metalloproteinase; PD: probing depth; CAL: clinical attachment level; EPA: eicosapentaenoic acid; DHA: docosahexaenoic acid; NSAIDs: non-steroidal anti-inflammatory drug; SRP: scaling and root planing. [10-14].

Agent	Principal target / mechanism	Evidence status in periodontitis
Subantimicrobial-dose doxycycline	Inhibits host-derived MMPs (collagenases)	Modest PD and CAL gains; low to very low certainty
Omega-3 PUFAs (EPA/DHA)	Anti-inflammatory and pro-resolving; also studied with low-dose aspirin	Potential PD/CAL improvement; variable, limited evidence
Specialized pro-resolving mediators	Actively promote resolution of inflammation	Emerging; mechanistic and early clinical support
NSAIDs	Inhibit cyclooxygenase and prostaglandin production	Anti-inflammatory but limited by systemic adverse effects
Bisphosphonates	Inhibit osteoclast-mediated bone resorption	Adjunctive benefit with SRP; systemic safety concerns
Probiotics / next-generation probiotics	Ecological modulation combined with immunomodulation	Modest adjunctive benefit; heterogeneous, short follow-up [8]

Next-generation probiotics and oral microbiome engineering

The oral cavity is a complex and organized system characterized by high microbial diversity and distinct environmental niches, in which the resident microbiota establishes ecological interactions that contribute to colonization resistance and the maintenance of homeostasis [17]. Targeted modulation of these interactions is therefore a plausible strategy to promote a healthy oral ecosystem [18]. Probiotics can act through mechanisms such as competitive inhibition and coaggregation with pathogenic microorganisms, potentially altering their adhesion and establishment within the oral biofilm [19]. Prebiotics are substrates that selectively stimulate resident microorganisms to favor microbial balance, synbiotics combine probiotic microorganisms with prebiotic substrates to support their functional activity, and postbiotics consist of inanimate preparations of microorganisms and their components, offering greater stability by not requiring viable organisms [19-21].

Probiotics can interfere with the establishment of disease-associated microorganisms through competition, adhesion, and interaction with the biofilm structure [20]. However, in vitro studies show that these capacities vary between probiotic strains [22], indicating that modulation of the oral ecosystem depends on the specific functional characteristics of each strain and supporting the importance of targeted probiotic selection [23]. For this reason, the development of next-generation probiotics has been promoted, in which strains are selected and oriented toward specific functions using tools such as microbiome analysis, genetics, bioinformatics, and synthetic biology [24]. The modification and selection of strains with directed functions has also begun to be studied against defined periodontal microbial targets [23-24], representing a shift from broad-spectrum microbial elimination toward a more specific ecological modulation of the subgingival microbiome [25].

Despite this potential, the heterogeneity of interventions and the limited follow-up periods make it difficult to determine whether the observed changes will last and contribute to stable ecological restoration [21]. Although next-generation probiotics represent a promising and innovative strategy, they still require greater validation of their safety and efficacy, together with improved standardization for clinical application [25].

Immunomodulatory effects of probiotics in periodontal disease

Beyond their ecological actions, probiotics exert immunomodulatory effects that are directly relevant to periodontal homeostasis; much of the mechanistic evidence derives from gut and systemic models and is extrapolated to the periodontal context, where it still requires site-specific confirmation. These effects may involve the regulation of innate and adaptive immune responses, including modulation of neutrophil activity, macrophage polarization, and inflammatory signaling pathways [26-27]. Probiotic supplementation has been associated with reduced levels of pro-inflammatory mediators, including IL-1β, TNF-α, IL-6, and IL-17, while potentially enhancing anti-inflammatory mechanisms such as IL-10 production and pathways involved in the resolution of inflammation [28-29]. In addition, probiotics may help reduce oxidative stress, improve epithelial barrier integrity, and influence mechanisms associated with osteoclast activity and alveolar bone remodeling [29-30]. By restoring a more balanced inflammatory environment, probiotics may contribute to the preservation of periodontal tissues and support healing following periodontal therapy, although further well-designed clinical and mechanistic studies are needed to determine optimal strains, dosage, duration, and long-term benefits [30].

Clinical evidence: probiotics as adjuncts to scaling and root planing

Scaling and root planing (SRP) remains the standard of non-surgical periodontal therapy. Because mechanical debridement alone may not fully address the microbial and inflammatory components of periodontitis, adjunctive therapies have been investigated to enhance bacterial reduction, reduce pocket depth, and improve control of inflammation, and probiotics have emerged as one such approach (Table 2) [31-32]. Current evidence suggests that probiotics may provide modest additional clinical benefits as adjuncts to SRP. A 2024 network meta-analysis of 33 randomized controlled trials involving 1,290 participants found improvements in probing pocket depth (PPD), CAL, and bleeding on probing (BOP) with several probiotic regimens, with Lactobacillus reuteri showing the most consistent favorable signal [33]. Similarly, a meta-analysis of 19 randomized trials reported approximately 0.60 mm greater PPD reduction and 0.52 mm greater CAL improvement with adjunctive probiotics compared with SRP alone or placebo, although substantial heterogeneity among strains, formulations, and protocols limits the generalizability of these findings [34].

**Table 2:** Representative clinical studies of probiotics as adjuncts to scaling and root planing in periodontitis. SRP: scaling and root planing; RCT: randomized controlled trial; PPD: probing pocket depth; CAL: clinical attachment level; BOP: bleeding on probing. [33-37].

Study (year)	Design	Probiotic / strain	Comparison	Key outcomes
Mendonça et al. [33]	Network meta-analysis (33 RCTs, 1,290 participants)	Various; L. reuteri most studied	SRP + probiotic vs SRP	Improved PPD, CAL, BOP; L. reuteri most consistent signal
Li et al. [34]	Systematic review and meta-analysis (19 RCTs)	Various	SRP + probiotic vs SRP or placebo	~0.60 mm greater PPD reduction, ~0.52 mm greater CAL gain; high heterogeneity
Jebin et al. [35]	Randomized placebo-controlled trial	Streptococcus salivarius UBSS-01	SRP + probiotic vs SRP + placebo	Greater 3-mo PPD reduction (1.74 vs 0.93 mm) and CAL gain; ↓ P. gingivalis, ↓ salivary IL-6
Vanditha et al. [36]	Randomized double-blind placebo-controlled trial	Lactobacillus reuteri (local fiber)	SRP + probiotic vs SRP + placebo	No significant additional PPD/CAL/BOP benefit at 3 months
Sufaru et al. [37]	Split-mouth randomized study	L. reuteri DSM 17938 (local)	SRP + probiotic vs SRP	Significant improvement in PPD, CAL, and BOP

While pooled analyses suggest an overall adjunctive benefit, individual trials reveal a less uniform response. A randomized placebo-controlled trial evaluating Streptococcus salivarius UBSS-01 reported significantly greater three-month PPD reduction (1.74 vs 0.93 mm) and CAL gain (1.02 vs 0.64 mm) with SRP plus probiotics, accompanied by greater reductions in P. gingivalis and salivary IL-6 [35]. Conversely, a randomized trial using locally delivered L. reuteri fibers found no significant additional improvement in PPD, CAL, or bleeding compared with SRP plus placebo at three months [36]. Other L. reuteri protocols have yielded positive results, with repeated local administration of L. reuteri DSM 17938 significantly improving PPD, CAL, and BOP compared with SRP alone [37]. Together, these findings indicate that the clinical response is not uniform and may vary according to strain, formulation, delivery method, treatment duration, and patient characteristics.

Beyond clinical parameters, evidence on microbiological and inflammatory outcomes remains limited and inconsistent. Some studies report reductions in selected pathogens, including P. gingivalis, while changes in inflammatory biomarkers vary across trials; reductions in salivary IL-6 have been observed with S. salivarius UBSS-01, whereas probiotic supplementation did not significantly reduce IL-1β in some populations [35]. Most trials have relatively short follow-up, and probiotic viability or detection tends to decline after administration, leaving the durability of these effects uncertain [32]. Taken together, probiotics may provide additional clinical benefits when used as adjuncts to SRP, although effectiveness remains inconsistent; differences in strains, dosages, delivery methods, durations, and patient characteristics make it difficult to establish a standardized protocol, and further well-designed trials with standardized protocols and longer follow-up are needed to determine clinical relevance and predictability [31].

Future perspectives: precision microbiome therapy and personalized periodontal medicine

Periodontitis is no longer plausibly framed as an infection to be eradicated, but as a breakdown of ecological and immunological homeostasis at the gingival interface. Keystone pathogens such as P. gingivalis subvert complement and innate immune signaling, polymicrobial synergy amplifies a dysbiotic community, and the resulting sustained host response, driven by IL-1β, TNF-α, IL-6, and IL-17, executed by MMP-8 and MMP-9, and translated into bone loss through RANKL/OPG imbalance, accounts for most attachment and osseous destruction [2,6]. Next-generation probiotics are conceptually attractive because they may act on both nodes at once: competing with and displacing pathobionts, restoring microbial diversity, stabilizing biofilm architecture and pH, and interfering with quorum sensing, while concurrently downregulating pro-inflammatory mediators, promoting IL-10 and regulatory T-cell responses, favoring M2 macrophage polarization, and reinforcing epithelial barrier integrity [18,30]. Microbiome engineering therefore differs from conventional antimicrobial therapy not in degree but in intent: reconstruction rather than elimination [25].

Looking forward, the most credible path is not stronger probiotics but smarter ones. Precision approaches, including rationally selected commensal consortia, engineered strains with defined effector functions, postbiotic and bacteriocin-based therapeutics, and community-editing strategies, together with multi-omics profiling and artificial-intelligence-driven prediction of individual dysbiotic signatures, point toward periodontal care matched to a patient's specific ecological and immunological phenotype rather than delivered uniformly [24-25]. Realizing this will require standardized strain-specific protocols, adequately powered long-term trials with post-intervention follow-up, harmonized outcome measures, and clear regulatory frameworks addressing safety, antimicrobial resistance gene transfer, and long-term ecological stability [25].

## Conclusions

Current-generation oral probiotics can meaningfully attenuate periodontal inflammation and enhance the outcomes of conventional therapy, but the clinical evidence supports a more measured conclusion than the biological rationale alone would predict. Used adjunctively to scaling and root planing, probiotics produce statistically significant but modest improvements in probing depth, clinical attachment level, and bleeding on probing, with the most reproducible benefits in deep pockets and in residual sites after non-surgical therapy. The microbial effect, however, is largely transient, colonization is rarely durable, and the literature remains heterogeneous in strain, dose, delivery, and follow-up. The honest answer to the central question of this review is therefore qualified: targeted microbiome modulation can help restore a more favorable inflammatory state, but present-generation probiotics do not independently reverse established periodontitis and do not displace mechanical disruption of the biofilm as the foundation of treatment. The trajectory of the evidence nonetheless supports a genuine reorientation of periodontal therapy, from broad-spectrum bacterial elimination toward ecological restoration and controlled host modulation, provided that standardized protocols, long-term trials, and clear safety frameworks are established.
